# Association Between Migraine and Frailty Among Middle-Aged and Older Adults: A Cross-Sectional Study Based on CHARLS

**DOI:** 10.1155/prm/8392878

**Published:** 2025-12-01

**Authors:** Shuoyu Rui, Zhilong Cai, Jingjing Wu, Jing Zhou, Fuying Liu, Nanqu Huang, Yong Luo, Fei Feng

**Affiliations:** ^1^Department of Neurology, Third Affiliated Hospital of Zunyi Medical University (The First People's Hospital of Zunyi), Zunyi, Guizhou, China; ^2^National Drug Clinical Trial Institution, Third Affiliated Hospital of Zunyi Medical University (The First People's Hospital of Zunyi), Zunyi, Guizhou, China

**Keywords:** Chinese population, frailty, Frailty Index, middle-aged and older adults, migraine

## Abstract

**Background:**

Frailty represents a geriatric syndrome characterized by diminished physiological reserves and increased vulnerability to adverse health outcomes. While chronic diseases are established frailty risk factors, the relationship between migraine—a prevalent neurological condition affecting millions globally—and frailty development remains unexplored, representing a critical knowledge gap in geriatric neurology.

**Methods:**

We conducted a cross-sectional analysis using baseline data (2011–2015) from the China Health and Retirement Longitudinal Study (CHARLS), a nationally representative cohort. Frailty was assessed using a validated 32-item Frailty Index based on deficit accumulation theory. Multivariable logistic regression models examined migraine-frailty associations, with sequential adjustment for sociodemographic factors, lifestyle behaviors, and comorbid conditions. Subgroup analyses evaluated effect modification across key demographic and clinical variables.

**Results:**

Among 13,798 participants (mean age 57.6 ± 9.1 years; 49.6% female), 598 (4.3%) reported migraine and 1,315 (9.5%) met frailty criteria. Migraine participants demonstrated a 4.5-fold higher frailty prevalence (37.0% vs. 8.3%, *p* < 0.001) and significantly elevated median frailty scores (20.0 vs. 7.8, *p* < 0.001). A multivariable analysis revealed a robust independent association (OR = 5.61, 95% CI: 4.51–6.99, *p* < 0.001). Smoking status significantly modified this relationship (P-interaction = 0.025), with the strongest associations in ever-smokers (OR = 8.78, 95% CI: 4.36–17.69) and current smokers (OR = 7.30, 95% CI: 4.87–10.96).

**Conclusion:**

This study establishes migraine as a novel independent risk factor for frailty in Chinese middle-aged and older adults. The pronounced smoking interaction suggests targeted tobacco cessation interventions may benefit frailty prevention in migraine patients.

## 1. Introduction

Migraine represents one of the most prevalent neurological disorders globally, characterized by recurrent moderate to severe headache episodes accompanied by nausea, vomiting, photophobia, and phonophobia [1]. As this is the second leading cause of disability worldwide, migraine affects over one billion individuals globally [2, 3]. Women experience approximately three-fold higher prevalence than men, particularly during reproductive years [2, 3]. While migraine prevalence generally declines with advancing age, a considerable proportion of older adults continue experiencing symptoms with persistent impacts on quality of life and healthcare utilization [4].

The pathophysiological mechanisms underlying migraine involve peripheral and central sensitization, deficient habituation, thalamocortical dysrhythmia, and motor cortex hyperexcitability [5–7]. The trigeminovascular system and the release of vasoactive neuropeptides such as calcitonin gene-related peptide (CGRP) play fundamental roles, resulting in neurogenic inflammation, vasodilation, and pain transmission [8, 9]. Additionally, migraine extends beyond a neurological disorder, demonstrating significant associations with cardiovascular diseases, stroke, mood disorders, and sleep disturbances [10, 11].

Frailty is a distinct geriatric syndrome marked by reduced physiological reserves and diminished ability to respond to stress, leading to increased vulnerability to adverse health outcomes [12]. The 32-item Frailty Index (FI-32), based on deficit accumulation theory, provides comprehensive assessment through continuous scoring (0–1) of health deficits, offering superior sensitivity for early frailty detection compared to phenotypic approaches [13]. Frailty affects 10%–15% of individuals aged ≥ 65 years, increasing to 25%–40% in those ≥ 80 years, and strongly predicts adverse outcomes including falls, hospitalization, disability, and mortality [14–17].

Recent genetic evidence has established bidirectional causal relationships between frailty and chronic pain, coexistence of both conditions exacerbates each other through shared pathophysiological mechanisms including Body Mass Index (BMI), smoking, physical inactivity, educational attainment, and depression [18]. Clinical intervention studies have shown that targeting geriatric syndromes can improve headache symptoms in older adults [19]. However, research specifically examining migraine-frailty associations remains limited, particularly in Asian populations.

This study addresses this gap by examining the migraine-frailty association using nationally representative cross-sectional data from the China Health and Retirement Longitudinal Study (CHARLS). We aimed to determine if migraine is associated with increased frailty risk after adjusting for confounders. Our findings offer novel insights into connections between neurological disorders and geriatric syndromes, potentially informing integrated clinical and preventive strategies.

## 2. Methods

### 2.1. Study Design and Participants

**Figure 1:**
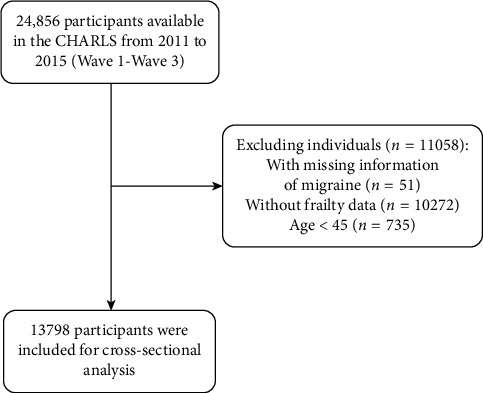
Flow diagram for participants included in the study.

This cross-sectional study utilized data from the CHARLS, a nationally representative survey designed to examine health and socioeconomic status among Chinese adults aged 45 years and above. The CHARLS employs a multistage probability sampling strategy covering 150 counties/districts and 450 villages/communities across 28 provinces in China, ensuring comprehensive geographic representation of the Chinese population. The national baseline survey was conducted between June 2011 and March 2012, with subsequent follow-up waves conducted in 2013, 2015, 2018, and 2020. To maintain sample representativeness over time, CHARLS replenishes the cohort biennially by adding newly eligible individuals (e.g., aging-in participants) through multistage stratified probability-proportional-to-size (PPS) sampling, consistent with the original baseline survey methodology. For the present analysis, we included baseline survey data spanning from 2011 to 2015. A total of 13,798 eligible participants (aged ≥ 45 years) meeting complete-data criteria were included in the final analytical dataset. Exclusion criteria were applied sequentially as follows: (1) participants with missing data on migraine status, (2) participants with missing data on Frailty Index components, and (3) participants aged < 45 years (Figure 1).

Data collection adhered to rigorous standardized protocols. Trained interviewers conducted structured face-to-face interviews using computer-assisted personal interviewing (CAPI) technology to minimize bias and ensure cross-site consistency. Concurrently, qualified health professionals performed physical examinations and anthropometric measurements following established protocols, guaranteeing data quality and reliability. This systematic approach encompassed comprehensive data across multiple domains—including sociodemographic characteristics, lifestyle and health behaviors, medical history, chronic disease status, and functional assessments—in strict accordance with the CHARLS study protocol, ensuring high-quality, standardized data suitable for epidemiological analysis.

### 2.2. The Definitions of Migraine

Migraine was operationally defined using criteria adapted from the validated ID Migraine Questionnaire, a widely used clinical screening tool to balance diagnostic accuracy with feasibility in large-scale surveys (17). The original ID Migraine demonstrates high sensitivity (0.81) and specificity (0.75) for identifying migraine according to ICHD criteria. It comprises three validated items: headache-related disability, nausea, and photophobia, with a positive screen defined as meeting at least two of these criteria [20]. Due to CHARLS questionnaire constraints, we utilized an adapted approach employing two specific survey items: “currently feeling stomach pain” and “currently feeling head pain.” These items correspond to the nausea and headache-related disability components of the original ID Migraine tool, respectively. For this study, migraine was defined as the concurrent presence of both head pain and stomach pain, representing a conservative adaptation of the established ID Migraine screening methodology [21]. This operational definition ensures specificity while maintaining alignment with validated migraine screening principles in the context of available CHARLS data. The adapted version retained the core discriminative items while enabling systematic screening by trained nonclinical interviewers across diverse literacy levels. Although it does not capture all migraine features (e.g., aura and attack duration), it maintains essential diagnostic components distinguishing migraine from other headache types, making it appropriate for examining migraine-health outcome associations in large-scale epidemiological research [22].

### 2.3. Assessment of Frailty

Frailty was assessed using a FI-32 based on deficit accumulation principles. Following systematic screening of the CHARLS dataset, we selected 32 variables encompassing comorbidities, physical function, disability, depression, and cognition (Table S1).

Variable coding: Items 1–31 were dichotomized (0 = *absence of deficit*, 1 = *presence of deficit*) based on predetermined cutoff values. The 32nd item was a continuous cognitive variable (range: 0–1), with higher values indicating greater cognitive impairment.

The FI-32 was calculated as (Sum of current health deficits ÷ 32) × 100, yielding a continuous variable ranging from 0 to 100. Higher values indicate greater frailty severity. Frailty was defined as FI-32 ≥ 25, consistent with established thresholds [23].

### 2.4. Covariates

We included multiple covariates known to be associated with both migraine and frailty based on the prior literature [24]. Sociodemographic factors included age, sex (male/female), education level (junior high school or below, high school, and higher education), marital status (married, widowed, and never married), and residence (urban/rural). Lifestyle factors comprised smoking status (never, former, and current), alcohol consumption (never, former, and current), and physical activity (no/yes). Health-related variables included BMI (calculated as weight in kilograms divided by height in meters squared), and self-reported physician-diagnosed chronic conditions (hypertension, diabetes, cancer, chronic lung disease, stroke, psychological diseases, rheumatoid arthritis, liver disease, kidney disease, digestive diseases, asthma, and memory-related diseases). Additional measures included social leisure activity (no/yes), depressive symptoms (assessed using the 10-item Center for Epidemiologic Studies Depression Scale), cognitive score (measured using a global cognitive score combining episodic memory, visuospatial abilities, orientation, and attention), and sensory impairments (visual and hearing impairment, each coded as no/yes). These covariates were assessed through standardized questionnaires and measurements during the CHARLS interviews and examinations [25].

### 2.5. Statistical Analysis

For descriptive statistics, continuous variables are expressed as mean ± standard deviation (SD) or median (interquartile range [IQR]). Categorical variables are presented as counts (percentages). The Kruskal–Wallis rank-sum test was used for continuous variables, and the chi-square test was used for categorical variables in the baseline characteristic analysis. This approach allows us to accurately and precisely analyze and compare different groups, enabling us to gather valuable insights and draw meaningful conclusions from our study.

To explore the relationship between migraine and frailty, we first conducted univariate regression analysis, followed by multivariable logistic regression models to analyze the relationship between migraine and frailty. Model 1 was a crude model that did not account for covariates; however, in Model 2, the analysis was adjusted for variables such as age, gender, education, and marital status. In Model 3, adjustments were made for residence, BMI, smoking, alcohol consumption, social activities, sleep duration, and physical activity based on Model 2. Additionally, we thoroughly examined interactions and stratifications based on various factors such as age, gender, BMI, smoking, alcohol consumption, physical activity, and sleep duration. To ensure the robustness of our findings, we performed sensitivity analyses. Missing covariate data were handled using multiple imputation with chained equations, creating five imputed datasets to account for uncertainty in the imputed values. All analyses were performed using R Statistical Software (Version 4.2.2, http://www.R-project.org, The R Foundation) [26]. *p* values less than 0.05 were considered to indicate statistical significance.

### 2.6. Ethics Statement

The CHARLS study received approval from Peking University's Biomedical Ethics Review Committee (IRB00001052-11015) and adhered to the Declaration of Helsinki's ethical standards [27]. All participants provided written informed consent prior to data collection. Our analysis utilized deidentified public-use datasets accessed with permission from the CHARLS research team. As we conducted the secondary analysis of anonymized data, no additional ethical approval was necessary. Our use of CHARLS data complies with all established data sharing and privacy protection policies of both the CHARLS research team and Peking University.

## 3. Results

### 3.1. Baseline Characteristics of the Study Population

Table 1 illustrates the demographic and clinical characteristics of this cross-sectional study, comprising 13,798 participants divided between migraine sufferers (*n* = 598) and nonmigraineurs (*n* = 13,200). Significant disparities emerge between these groups, with migraine patients exhibiting a predominance of female participants (70.2% vs. 48.7%, *p* < 0.001), lower educational attainment and rural residence. Furthermore, the migraine cohort demonstrated significantly elevated frailty scores (median 32-FI: 20.0 vs. 7.8) and prevalence (37.0% vs. 8.3%, *p* < 0.001) (Table 1).

### 3.2. The Relationships Between Migraine and Frailty

Table 2 presents the results of the multivariable logistic regression analysis, where we adjusted for three models, and all results consistently indicate a positive correlation between migraine and frailty. The data demonstrate that individuals with migraine exhibit a substantially higher prevalence of frailty (OR = 5.61,95% CI: 4.51–6.99, *p* < 0.001) after comprehensive adjustment for potential confounding variables. The minimal attenuation of effect size from the unadjusted model (OR = 6.49) to the fully adjusted model suggests that this association exists largely independent of sociodemographic factors, comorbidities, and lifestyle characteristics. These findings highlight migraine as a potentially significant independent risk factor for frailty, which may have important implications for clinical screening and preventive interventions (Table 2).

### 3.3. Sensitivity Analysis

The sensitivity analyses conducted to examine the association between migraine and frailty demonstrate a robust and significant relationship across increasingly stringent exclusion criteria. Initially, after excluding cases with missing covariate data, individuals with migraine exhibited substantially higher frailty prevalence (36.8%) compared to nonmigraineurs (8.2%), with an adjusted odds ratio of 5.61 (95% CI: 4.51–6.99) (Table S2). This association persisted when excluding participants with gastrointestinal diseases (OR = 5.74, 95% CI: 4.03–8.18) and strengthened further when both gastrointestinal diseases and arthritis were excluded (OR = 8.18, 95% CI: 4.71–14.22) (Tables S3 and S4). The consistency of these findings across multiple analytical approaches suggests a genuine biological connection rather than mere statistical confounding, highlighting the potential clinical significance of migraine as a risk indicator for frailty, particularly in older populations.

### 3.4. Subgroup Analysis and Interaction Effects

**Figure 2:**
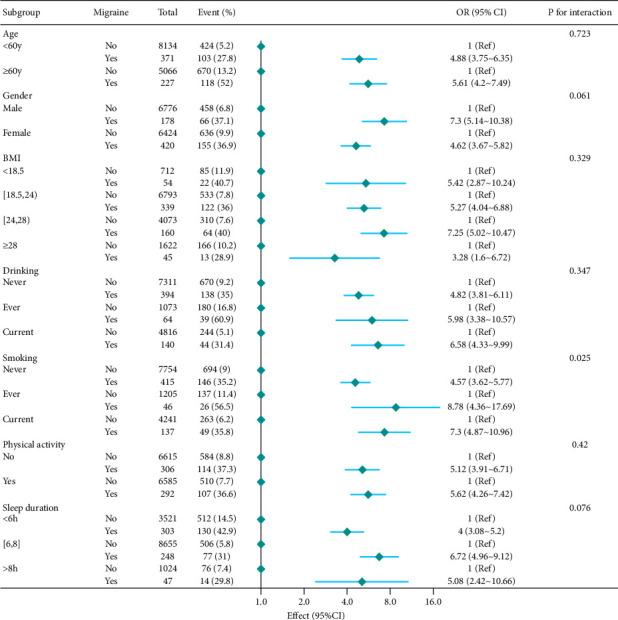
Subgroup analysis and interaction of the association between migraine and frailty. OR, odds ratio; CI, confidence interval.

Figure 2 reveals a robust association between migraine and frailty across various subgroups, remaining significant in both younger (< 60 years, OR = 4.88) and older adults (≥ 60 years, OR = 5.61; *P*-interaction = 0.723). A borderline gender interaction was observed, with a stronger association in males (OR = 7.3) than in females (OR = 4.62; *P*-interaction = 0.061). Smoking status demonstrated the most pronounced effect modification, with ever-smokers (OR = 8.78) and current smokers (OR = 7.3) showing higher risks compared to never-smokers (OR = 4.57; *P*-interaction = 0.025), while sleep duration, BMI, alcohol use, and physical activity showed no significant interactions. These findings highlight the need to address migraines and modifiable factors, particularly smoking, in frailty evaluations (Figure 2).

## 4. Discussion

In this large, nationally representative cross-sectional study of 13,798 middle-aged and older adults from the CHARLS cohort, we identified a significant association between migraine and frailty. After adjusting for a comprehensive range of potential confounders, individuals with migraine had more than five-fold higher odds of frailty compared to those without migraine. Sensitivity analyses, including exclusion of participants with gastrointestinal diseases and arthritis, further support the robustness of this association. Subgroup analyses suggest this relationship persists across age strata and is notably pronounced among current and former smokers. These findings reveal migraine as a potentially underrecognized risk factor for frailty, underscoring the need for clinicians to consider frailty screening in the middle-aged and older population.

Although migraine has been widely acknowledged as a contributor to various adverse health outcomes, its specific relationship with frailty remains largely unexplored. Substantial literature has documented associations between chronic pain conditions and frailty in older adults. A recent meta-analysis demonstrated that persons with chronic pain were 1.85 times more likely to develop frailty after an average follow-up of 5.8 years [28]. Similarly, longitudinal studies have confirmed that chronic widespread pain significantly increases frailty risk in community-dwelling older men [29], while several studies have shown strong associations between pain, cognitive function, and frailty components [30, 31]. However, migraine-specific research in this context has been limited to fatigue-related outcomes, such as Hsu et al. found that migraine patients had a 1.5-fold higher risk of developing chronic fatigue syndrome [32].

The focus on middle-aged and older adults (≥ 45 years) in our study holds particular clinical significance. Although migraine prevalence generally peaks during reproductive years, the condition continues to impose substantial burden on older populations, with studies reporting persistent migraine in 10%–20% of adults aged ≥ 60 years [33]. Importantly, our subgroup analyses demonstrated consistent migraine-frailty associations across age strata (< 60 years: OR = 4.88; ≥ 60 years: OR = 5.61; P-interaction = 0.723), suggesting that this relationship reflects shared pathophysiological mechanisms warranting clinical attention throughout the aging process. Moreover, the implications of our findings may extend to broader age ranges. Given that frailty represents a cumulative deficit accumulation process over the life course [34], and considering the bidirectional causal relationship between chronic pain and frailty demonstrated by recent Mendelian randomization studies, migraine exposure at younger ages may initiate trajectories toward accelerated frailty development in later life. The pronounced smoking interaction identified in our study (P-interaction = 0.025) further underscores the importance of early preventive interventions. Future longitudinal studies should investigate whether migraine management in younger adults can mitigate long-term frailty risk, informing life-course approaches to integrated neurological and geriatric care across the lifespan.

In addition, our findings extend this knowledge base by providing the first large-scale evidence specifically linking migraine to comprehensive frailty assessment. Unlike previous studies focusing on individual frailty components such as fatigue or weakness, our research employed a validated multidimensional Frailty Index that captures the full spectrum of age-related deficits. The observed effect size (OR = 5.61) substantially exceeds those reported for general chronic pain conditions, suggesting that migraine may confer particularly high frailty risk. Furthermore, our identification of smoking as a significant effect modifier aligns with emerging evidence of shared inflammatory pathways between pain, smoking, and frailty [35]. These findings position migraine as a potentially underrecognized but clinically significant predictor of frailty, warranting inclusion in comprehensive geriatric assessments.

The ID Migraine Questionnaire, a validated three-item screening tool assessing headache with activity limitation, nausea, and photophobia, demonstrates 81% sensitivity and 75% specificity for migraine diagnosis [20]. This approach makes our findings particularly suitable for large-scale epidemiological studies and routine clinical screening, as the questionnaire is brief, cost-effective, and can be easily implemented in primary care settings. Compared to the gold-standard International Classification of Headache Disorders (ICHD-3) criteria, the ID Migraine Questionnaire cannot differentiate between migraine subtypes (migraine with or without aura) or assess attack frequency and duration, which may have clinical relevance for frailty risk stratification [36]. Therefore, future research employing ICHD-3 criteria should explore whether specific migraine subtypes, attack patterns, or severity measures demonstrate differential associations with frailty components. Such investigations could inform more personalized risk stratification and targeted intervention strategies for migraine patients at heightened frailty risk.

The biological mechanisms underlying the migraine-frailty association are multifaceted. First, inflammatory processes represent the central shared pathway. Both conditions exhibit elevated proinflammatory cytokines (IL-6, TNF-α, and CRP) [37–39]. Migraine attacks trigger neurogenic inflammation through CGRP and substance P release [8, 9], potentially evolving into persistent systemic inflammation that aligns with “inflammaging” in frailty pathophysiology [40]. Second, neurovascular and metabolic dysfunction provide additional links. Migraine features persistent cerebrovascular dysregulation and endothelial dysfunction [41], contributing to microvascular compromise that impairs tissue perfusion—a key frailty mechanism [39]. Both conditions demonstrate mitochondrial dysfunction and oxidative stress [42, 43], with recent metabolomic analyses identifying overlapping metabolic signatures between migraine and aging pathways. Third, behavioral consequences further contribute to frailty risk. Pain-related disability reduces physical activity, promoting deconditioning and sarcopenia [44]. The psychological burden may compromise stress response systems through neuroendocrine dysregulation [45]. In addition, the gut–brain axis represents an emerging pathway, with altered microbiota documented in both conditions potentially triggering systemic inflammation [46, 47]. Future longitudinal studies with repeated assessments are needed to establish temporal relationships and clarify directionality. Mechanistic investigations employing biomarker profiling (inflammatory cytokines and neuroimaging markers) in migraine patients across the aging spectrum could elucidate specific pathways. Additionally, trials examining whether effective migraine management—including CGRP-targeted therapies—can modify frailty trajectories would provide critical evidence for causality and inform integrated preventive strategies.

While our study provides robust evidence for the migraine-frailty association in Chinese adults, generalizability to other populations warrants consideration. Migraine prevalence varies substantially (8%–12% in Asia vs. 12%–18% in Western populations), attributed to genetic polymorphisms, environmental triggers, and diagnostic practices (2, 45, 46). Similarly, frailty prevalence ranges from 4% to 59% globally, with marked phenotypic and cultural differences between populations (47, 48). Cultural and lifestyle factors may serve as important effect modifiers. Dietary differences—such as higher fish and green tea consumption in Asian diets—may confer neuroprotective effects that attenuate migraine-related neuroinflammation (49, 50). Physical activity, smoking, and alcohol consumption also show cross-cultural variation (51). Additionally, social support systems and healthcare-seeking behaviors differ between Eastern and Western cultures, potentially influencing both migraine management and frailty progression (52, 53). Future international collaborative studies using standardized protocols are needed to verify these findings across diverse populations.

Several critical limitations warrant consideration. First, migraine assessment relied on self-reported CHARLS symptoms rather than standardized clinical diagnosis. While these align with validated ID Migraine screener components, they lack the precision of formal ICHD-3 criteria, although sensitivity analyses excluding gastric disorders supported association robustness. Future studies incorporating more comprehensive tools (e.g., full ICHD-3 criteria or MIDAS questionnaire) could examine dose–response relationships and whether specific migraine phenotypes confer differential frailty risk, informing more targeted preventive strategies. Second, the cross-sectional design precludes causal inference. Third, residual confounding from unmeasured factors (genetics, medications, and inflammatory markers) likely persists despite extensive adjustment. Finally, although our hypothesis-driven analysis focused on one primary exposure-outcome relationship with prespecified subgroups, we did not apply formal multiple comparison corrections, and the possibility of false-positive findings in subgroup analyses cannot be entirely excluded. Future studies specifically examining smoking as an effect modifier are warranted to confirm this interaction and elucidate underlying mechanisms.

## 5. Conclusion

This large-scale population-based study demonstrates a strong independent association between migraine and frailty among Chinese middle-aged and older adults (adjusted OR = 5.61, 95% CI: 4.51–6.99, *p* < 0.001). The relationship was particularly pronounced among smokers, suggesting important effect modification by smoking status. This finding provides important evidence for early intervention against frailty in clinical practice for migraine patients. The mechanisms linking migraine and muscle function are not yet clear, future longitudinal studies with biomarker assessments are needed to establish temporal relationships and elucidate underlying mechanisms linking migraine to frailty development.

## Figures and Tables

**Table 1:** Characteristics of individuals according to migraine status.

Variables	Total (*n* = 13,798)	Nonmigraine (*n* = 13,200)	Migraine(*n* = 598)	*p* value
Age, mean ± SD	57.6 ± 9.1	57.6 ± 9.2	57.5 ± 8.3	0.766
Gender, *n* (%)				< 0.001
Male	6954 (50.4)	6776 (51.3)	178 (29.8)	
Female	6844 (49.6)	6424 (48.7)	420 (70.2)	
Education, *n* (%)				< 0.001
Junior high school and below	11,759 (85.2)	11,187 (84.8)	572 (95.7)	
High school and career training	1672 (12.1)	1647 (12.5)	25 (4.2)	
Higher education	367 (2.7)	366 (2.8)	1 (0.2)	
Marital status, *n* (%)				0.74
Married	12,218 (89.7)	11,690 (89.7)	528 (89.3)	
Widowed	1288 (9.5)	1231 (9.5)	57 (9.6)	
Unmarried	111 (0.8)	105 (0.8)	6 (1)	
Residence, *n* (%)				< 0.001
Urban	3666 (26.6)	3597 (27.3)	69 (11.6)	
Rural	10,120 (73.4)	9592 (72.7)	528 (88.4)	
BMI, median (IQR)	23.3 (21.0, 25.9)	23.3 (21.1, 25.9)	22.6 (20.2, 25.1)	< 0.001
Drinking, *n* (%)				< 0.001
Never	7703 (55.8)	7309 (55.4)	394 (65.9)	
Ever	1136 (8.2)	1072 (8.1)	64 (10.7)	
Current	4954 (35.9)	4814 (36.5)	140 (23.4)	
Smoking, *n* (%)				< 0.001
Never	8167 (59.2)	7752 (58.7)	415 (69.4)	
Ever	1251 (9.1)	1205 (9.1)	46 (7.7)	
Current	4378 (31.7)	4241 (32.1)	137 (22.9)	
Hypertension (%)				0.34
No	10,312 (74.7)	9875 (74.8)	437 (73.1)	
Yes	3486 (25.3)	3325 (25.2)	161 (26.9)	
Diabetes (%)				0.171
No	12,921 (93.6)	12,369 (93.7)	552 (92.3)	
Yes	877 (6.4)	831 (6.3)	46 (7.7)	
Cancer (%)				0.51
No	13,671 (99.1)	13,077 (99.1)	594 (99.3)	
Yes	127 (0.9)	123 (0.9)	4 (0.7)	
Chronic lung disease, *n* (%)				< 0.001
No	12,521 (90.7)	12,020 (91.1)	501 (83.8)	
Yes	1277 (9.3)	1180 (8.9)	97 (16.2)	
Heart disease, *n* (%)				< 0.001
No	12,141 (88.0)	11,666 (88.4)	475 (79.4)	
Yes	1657 (12.0)	1534 (11.6)	123 (20.6)	
Stroke, *n* (%)				0.504
No	13,505 (97.9)	12,922 (97.9)	583 (97.5)	
Yes	293 (2.1)	278 (2.1)	15 (2.5)	
Psychological diseases, *n* (%)				< 0.001
No	13,677 (99.1)	13,095 (99.2)	582 (97.3)	
Yes	121 (0.9)	105 (0.8)	16 (2.7)	
Rheumatoid arthritis, *n* (%)				< 0.001
No	9598 (69.6)	9361 (70.9)	237 (39.6)	
Yes	4200 (30.4)	3839 (29.1)	361 (60.4)	
Dyspepsia, *n* (%)				0.463
No	12,164 (89.0)	11,641 (89)	523 (88)	
Yes	1508 (11.0)	1437 (11)	71 (12)	
Liver disease, *n* (%)				< 0.001
No	13,296 (96.5)	12,749 (96.7)	547 (91.9)	
Yes	483 (3.5)	435 (3.3)	48 (8.1)	
Kidney disease, *n* (%)				< 0.001
No	13,015 (94.5)	12,515 (95)	500 (83.9)	
Yes	758 (5.5)	662 (5)	96 (16.1)	
Digestive disease, *n* (%)				< 0.001
No	10,873 (78.9)	10,642 (80.7)	231 (38.8)	
Yes	2915 (21.1)	2550 (19.3)	365 (61.2)	
Asthma, *n* (%)				< 0.001
No	13,273 (96.2)	12,722 (96.4)	551 (92.1)	
Yes	525 (3.8)	478 (3.6)	47 (7.9)	
Memory-related disease, *n* (%)				< 0.001
No	13,642 (98.9)	13,060 (98.9)	582 (97.3)	
Yes	156 (1.1)	140 (1.1)	16 (2.7)	
Social leisure activity, *n* (%)				0.001
No	6749 (48.9)	6418 (48.6)	331 (55.4)	
Yes	7048 (51.1)	6781 (51.4)	267 (44.6)	
CESD10, median (IQR)	6.0 (3.0, 11.0)	6.0 (3.0, 11.0)	14.0 (9.0, 18.0)	< 0.001
Cognition score, mean ± SD	12.5 ± 3.3	12.5 ± 3.3	10.8 ± 3.5	< 0.001
Sleep duration, mean ± SD	6.4 ± 1.8	6.5 ± 1.8	5.6 ± 2.2	< 0.001
Physical activity, *n* (%)				0.906
No	5877 (50.0)	5606 (50)	271 (50.3)	
Yes	5870 (50.0)	5602 (50)	268 (49.7)	
Visual impairment, *n* (%)				< 0.001
No	13,082 (94.8)	12,549 (95.1)	533 (89.1)	
Yes	716 (5.2)	651 (4.9)	65 (10.9)	
Hearing impairment, *n* (%)				< 0.001
No	12,898 (93.5)	12,375 (93.8)	523 (87.5)	
Yes	900 (6.5)	825 (6.2)	75 (12.5)	
FI-32, median (IQR)	7.9 (4.4, 14.5)	7.8 (4.2, 14.2)	20.0 (12.9, 29.5)	< 0.001
Frailty, *n* (%)				< 0.001
Nonfrailty	12,483 (90.5)	12,106 (91.7)	377 (63)	
Frailty	1315 (9.5)	1094 (8.3)	221 (37)	

Abbreviation: CESD10, Center for Epidemiologic Studies Depression Scale 10.

**Table 2:** Multivariable logistic regression analysis of the association between migraine and frailty after multiple imputation.

Variable	*n*. Total	*n*. Event_%	Model 1		Model 2		Model 3	
			OR (95 CI)	*P*	OR (95% CI)	*P*	OR (95% CI)	*P*
Nonmigraine	13,200	1094 (8.3)	1 (Ref)		1 (Ref)		1 (Ref)	
Migraine	598	221 (37)	6.49 (5.43–7.74)	< 0.001	6.31 (5.23–7.61)	< 0.001	5.61 (4.51–6.99)	< 0.001

*Notes:* Model 1 analysis was nonadjusted. Model 2 analysis was adjusted for age, gender, education level, and marital status. Model 3 analysis was additionally adjusted for residence, BMI, drinking, smoking, social leisure activity, sleep duration, and physical activity.

Abbreviations: OR, odd ratio; CI, confidence interval.

## Data Availability

The raw data required to reproduce these findings cannot be shared at this time, as the data also form part of an ongoing study. If necessary, some or all the data generated or used during the study are available from the corresponding authors upon request.

## Supporting Information

Table S1: The 32 items used to construct the Frailty Index.

Table S2: The association between migraine and frailty after removing all missing values from the variables.

Table S3: The association between migraine and frailty after removing gastrointestinal diseases.

Table S4: The association between migraine and frailty after removing gastrointestinal diseases and arthritis.
