# Supplementary material for: Association Between Migraine and Frailty Among Middle-Aged and Older Adults: A Cross-Sectional Study Based on CHARLS
Source: Pain Res Manag. 2025 Dec 1;2025:8392878. doi: 10.1155/prm/8392878 (PMC12685432; doi:10.1155/prm/8392878)
Supplement: Supporting Information — Additional supporting information can be found online in the Supporting Information section. [file 8392878.f1.docx]

**Supplementary material**

**Table S1.** The 32 items used to construct the frailty index

| No | Description of the items in the CHARLS | | Cut-off value |
| --- | --- | --- | --- |
|  |  |  |  |
| 1 | Self-reported physician diagnosed hypertension | | Yes = 1, No = 0 |
| 2 | Self-reported physician diagnosed diabetes | | Yes = 1, No = 0 |
| 3 | Self-reported physician diagnosed heart disease | | Yes = 1, No = 0 |
| 4 | Self-reported physician diagnosed stroke | | Yes = 1, No = 0 |
| 5 | Self-reported physician diagnosed cancer | | Yes = 1, No = 0 |
| 6 | Self-reported physician diagnosed arthritis | | Yes = 1, No = 0 |
| 7 | Self-reported physician diagnosed chronic lung disease | | Yes = 1, No = 0 |
| 8 | Self-reported physician diagnosed asthma | | Yes = 1, No = 0 |
| 9 | Self-reported physician diagnosed any emotional, nervous, or psychiatric problems | | Yes = 1, No = 0 |
| 10 | Self-reported physician diagnosed memory-related disease | | Yes = 1, No = 0 |
| 11 | Self-reported vision problems | Self-reported eyesight (while using lenses if appropriate) | Yes = 1, No = 0 |
| 12 | Self-reported hearing problems | Self-reported hearing (while using hearing aid if appropriate) | Yes = 1, No = 0 |
| 13 | Self-reported general health status | | Poor or very poor = 1, fair, very good, or good = 0 |
| 14 | Difficulty with dressing | | Yes = 1, No = 0 |
| 15 | Difficulty with bathing or showering | | Yes = 1, No = 0 |
| 16 | Difficulty with eating | | Yes = 1, No = 0 |
| 17 | Difficulty with getting in and out of bed | | Yes = 1, No = 0 |
| 18 | Difficulty with using the toilet | | Yes = 1, No = 0 |
| 19 | Difficulty with managing money | | Yes = 1, No = 0 |
| 20 | Difficulty with taking medications | | Yes = 1, No = 0 |
| 21 | Difficulty with shopping for groceries | | Yes = 1, No = 0 |
| 22 | Difficulty with preparing meals | | Yes = 1, No = 0 |
| 23 | Difficulty with doing housework | | Yes = 1, No = 0 |
| 24 | Mobility: difficulty with walking 100 yards | | Yes = 1, No = 0 |
| 25 | Mobility: difficulty with getting up from a chair after sitting for long periods | | Yes = 1, No = 0 |
| 26 | Mobility: difficulty with climbing several flights of stairs without resting | | Yes = 1, No = 0 |
| 27 | Mobility: difficulty with lifting or carrying weights over 10 pounds/jins | | Yes = 1, No = 0 |
| 28 | Mobility: difficulty with picking up a coin from the table | | Yes = 1, No = 0 |
| 29 | Mobility: difficulty with stooping, kneeling, or crouching | | Yes = 1, No = 0 |
| 30 | Mobility: difficulty with reaching arms above shoulder level | | Yes = 1, No = 0 |
| 31 | Depression: CESD-10 questionnaire | Depression: CESD-8 questionnaire | CESD-10 >10 =1, ≤10 =0 |
| 32 | Cognition: (memory test score + orientation test score) **/** 14 | | Continuous, ranging from 0 to 1 |

Heart disease indicates the angina, coronary heart disease, congestive heart failure, or other heart problems.

Memory-related disease indicates Alzheimer’s disease or dementia, organic brain senility, or other serious memory impairment.

Depression is evaluated using Center for Epidemiologic Studies Depression Scale (CESD). In the CHARLS, CESD-10 is used, and the total score ranges from 0 to 30. The higher score indicates more severe depressive symptoms.

The memory score is the average of words that are not recalled in the immediate and delayed word recall tasks. The memory score ranges from 0 to 10. The orientation test comprises four questions about the day of the week, the month, the date of the month, and the year. One point is given for each wrong answer, and the range is from 0 to 4.

32 - FI = Total Score / (32) * 100

Frailty was defined as the 32-FI ≥ 25

**Table S2.** The association between migraine and frailty after removing all missing values from the variables.

| **Variable** | **n.total** | **n.event_%** | **Model 1** | | **Model 2** | |
| --- | --- | --- | --- | --- | --- | --- |
|  |  |  | **OR (95CI)** | ***P*** | **OR (95%CI)** | ***P*** |
| Non-migraine | 9291 | 760 (8.2) | 1(Ref) |  | 1(Ref) |  |
| Migraine | 462 | 170 (36.8) | 6.54 (5.33~8.01) | <0.001 | 5.61 (4.51~6.99) | <0.001 |

OR = Odd ratio, CI = confidence interval.

Model 1 analysis was non-adjusted. Model 2 analysis was adjusted for age, gender, education level, marital status,residence,bmi,drinking,smoking, social leisure activity,sleep duration,physical activity

**Table S3.** The association between migraine and frailty after removing gastrointestinal diseases

| **Variable** | **n.total** | **n.event_%** | **Model 1** | | **Model 2** | |
| --- | --- | --- | --- | --- | --- | --- |
|  |  |  | **OR (95CI)** | ***P*** | **OR (95%CI)** | ***P*** |
| Non-migraine | 10642 | 751 (7.1) | 1(Ref) |  | 1(Ref) |  |
| Migraine | 231 | 73 (31.6) | 6.09 (4.57~8.11) | <0.001 | 5.74 (4.03~8.18) | <0.001 |

OR = Odd ratio, CI = confidence interval.

Model 1 analysis was non-adjusted. Model 2 analysis was adjusted for age, gender, education level, marital status,residence,bmi,drinking,smoking, social leisure activity,sleep duration,physical activity

**Table S4.** The association between migraine and frailty after removing gastrointestinal diseases and arthritis

| **Variable** | **n.total** | **n.event_%** | **Model 1** | | **Model 2** | |
| --- | --- | --- | --- | --- | --- | --- |
|  |  |  | **OR (95CI)** | ***P*** | **OR (95%CI)** | ***P*** |
| Non-migraine | 7930 | 311 (3.9) | 1(Ref) |  | 1(Ref) |  |
| Migraine | 115 | 24 (20.9) | 6.46 (4.06~10.27) | <0.001 | 8.18 (4.71~14.22) | <0.001 |

OR = Odd ratio, CI = confidence interval.

Model 1 analysis was non-adjusted. Model 2 analysis was adjusted for age, gender, education level, marital status,residence,bmi,drinking,smoking, social leisure activity,sleep duration,physical activity
